# Molecular epidemiology of human adenoviruses in children with and without respiratory symptoms: Preliminary findings from a case-control study

**DOI:** 10.1186/s12887-022-03625-3

**Published:** 2022-10-08

**Authors:** Sevrin Zadheidar, Jila Yavarian, Zahra Heydarifard, Ahmad Nejati, Kaveh Sadeghi, Nastaran Ghavami, Simin Abbasi, Somayeh Shatizadeh Malekshahi, Talat Mokhtari-Azad, Nazanin-Zahra Shafiei-Jandaghi

**Affiliations:** 1 Virology Department, School of Public Health, Tehran University of Medical Sciences, Tehran, Iran; 2 Department of Virology, Faculty of Medical Sciences, Tarbiat Modares University, Tehran, Iran

**Keywords:** Adenovirus, Genotyping, HAdV-B, Children, Respiratory infections

## Abstract

**Background:**

Human adenovirus (HAdV) is an important viral agent in children which can lead to severe acute respiratory infection (SARI). Reports on molecular epidemiology of HAdVs in Iran are limited. This case-control study is conducted to compare the HAdV infection rate and molecular epidemiology among two groups of children with and without respiratory symptoms in Tehran, Iran during 2018–2019.

**Methods:**

Nested PCR was performed on 120 oropharyngeal swabs taken from children aged five and younger with SARI who were hospitalized as the case group, and 120 oropharyngeal swabs were collected from children of the same age without respiratory symptoms as the control group. For positive samples Sanger sequencing was done and a phylogenetic tree was drawn afterward.

**Results:**

Out of 120 cases, 8 (6.6%) tested positive for eachHAdV types including 6 (75%) HAdV-B7, 1 (12.5%) HAdV-C2, and 1 (12.5%) HAdV-C6. Among the control group, out of 120 samples, 8 (6.6%) were positive comprising 5 (62.5%) HAdV-C5, 2 (25%) HAdV-F41, and 1 (12.5%) HAdV-C6.

**Conclusion:**

The present study indicated a different viewpoint of HAdV molecular epidemiology in which the genotypes were compared in children with and without respiratory symptoms. HAdV prevalence was equally common in cases and controls but different genotypes were detected in these two groups. HAdV-B7 was the main type among children with SARI, dissimilar to children with no respiratory symptoms where HAdV-C5 was the predominant type. Detecting HAdV-F in oropharyngeal swabs was a rare finding, which requires further investigation.

## Introduction

Human adenoviruses (HAdVs) are non-enveloped double-stranded linear DNA viruses belonging to the family *Adenoviridae* and genus Mastadenovirus. HAdVs are classified into seven species (A to G) and more than 90 types, due to their genetic characteristics, tissue tropism, and clinical manifestations. HAdV species C (type 1, 2, 5, and 6) and B (type 3 and 7) are most commonly related to respiratory infections, followed by species E and D, while viruses in species F are assumed to cause gastrointestinal tract infections[1]. The predominant HAdV types detected in association with clinical presentation differ between different geographic regions and may shift over time according to the year of surveillance. HAdVs are crucial viral agents which have been linked to a variety of illnesses, including severe acute respiratory infection (SARI), gastroenteritis, conjunctivitis, hemorrhagic cystitis, hepatitis, myocarditis, nephritis, meningoencephalitis, and nosocomial infection [2]. SARIs (of any viruses) are considered to be serious threats in pediatric patients [2–4]. HAdVs as one of the causative agents of SARI can involve all ages, however, their infections occur more frequently in children younger than 5 years old [2]. It is worth mentioning that SARI etiology can be hard to establish due to the frequent detection of HAdV in asymptomatic children [5, 6]. HAdVs account for 5–10% of respiratory tract infections among children [3, 7]. Clinical manifestations of adenoviral respiratory disease may range from asymptomatic infection to fatal [1, 4]. The severity of clinical symptoms depends on the host’s age and immunocompetence status as well as the types of the virus [2, 8]. Almost 80% of HAdV infections in children are related to HAdV types 1 to 7 [9]. HAdV-B7 is highly correlated with severe respiratory infections and high mortality rate [9–11]. Since respiratory HAdVs prodominent types alter.

through the years in various countries, evaluation of molecular epidemiology is necessary to recognize the prevalence of circulating HAdVs types in a specific period.

This case-control study is conducted to estimate the HAdV infection rate and its molecular epidemiology in children aged five and younger with respiratory symptoms (as case group) and without respiratory symptoms (as control group) from December 2018 to December 2019.

### Method

#### Study design and samples preparation

A total of 240 oropharyngeal specimens, collected from December 2018 to December 2019, were enrolled in this case-control study, including 120 oropharyngeal swabs from children aged five and younger hospitalized with SARI and referred to the National Influenza Center (NIC), all of which tested negative for influenza A and B viruses, and 120 oropharyngeal swabs which were collected from children with the same age group who had fever and rash due to MMR (Measles, Mumps and Rubella) vaccine and had no respiratory symptoms as the control group. All cases were individually matched to control by age. The presented study was approved by the Ethics Committee of Tehran University of Medical Sciences with the approval code IR.TUMS.SPH.REC.1398.162.

#### DNA extraction and PCR

DNA extraction was performed using the High Pure Viral Nucleic acid kit (Roche, Germany) according to the manufacturer’s instructions. Nested polymerase chain reaction (PCR) was performed on a hexon conserved region by applying specific primers including, ADH-01 (ACTACAAYATTGGCTACCAGG) and ADH-02 (CAAAACATAAAGAAGKGTGGGC) with 440bp PCR product for the first round and ADH-I1 (AACTTCCAGCCCATGAGCMG) and ADH-I2 (CTCAAAAGTCATGTCBAGCGC) with 330bp PCR product for the second round [12].

The first round of nested PCR was performed in a 50µl reaction. 10µl of extracted DNA was added to 40µl of the reaction mixture containing 5µl 10× PCR buffer,

20 mM MgSo4, 2.5 mM dNTP mixture, 2 units of *Taq* DNA polymerase (Biobasic, Canada) and 10 pmol of the ADH-01 and ADH-02 primers. The thermal cycling was carried out with the following program: initial denaturation at 94°C for 5min, followed by 40 cycles consisting of denaturation at 94°C for 30s, annealing at 50°C for 30s and extension at 72°C for 30s. The final elongation step was 72°C for 5mins. In the second round of PCR, 5µl of the primary PCR product was added to 45µl of the reaction mixture using ADH-I1 and ADH-I2 primers. The thermal protocol was the same as mentioned in the first round, apart from the annealing temperature which exceeded to 55°C. The final PCR products (330bp) were observed using 2% agarose gel electrophoresis and sequenced by Sanger sequencing in NIC laboratory using 3130 genetic analyzer (Applied Biosystems).

#### Phylogenetic analysis

The sequence reads were processed using BioEdit (v 7.0.5.3). The final sequences of each read were aligned by the BLAST Tool from NCBI. To draw a phylogenetic tree MEGA X was used based on the Neighbor-joining method, Tamura-Nei model, and bootstrap analyses by 1000 resampling of the datasets.

### Statistical analysis

Statistical analyses were done using R version 4.0.3 and SPSS software 16.0 (SPSS Inc, Chicago, IL).

To compare differences between children with and without respiratory symptoms, we used the χ2 test, or McNemar’s exact test were appropriate. A two-sided α value of less than 0.05 was regarded as statistically significant.

## Results

The specimens were oropharyngeal swabs from two groups of children aged five and younger with and without any respiratory symptoms as case and control groups, respectively. Each group was divided into 3 age groups: under 1, 1–3 and 4–5 years old. All cases in these two groups were individually matched by age. Hence, age distribution was almost identical in both groups comprising 56 (46.7%) under 1, 54 (45%) 1–3 and 10 (8.3%) 4–5 years old with a mean age of 1.05 years (SD = 1.38). Totally, out of 240 samples 16 (6.6%) were positive for HAdV by nested PCR including 8 (6.7%) positive in the patients and 8 (6.7%) positive in the control group. Out of all 120 individuals in the control group, 51 (42.5%) were female and 69 (57.5%) were male containing 4 (50%) positive females and 4(50%) positive males. 47 (39.1%) of 120 patients were female and 73 (60.9%) were male comprising 5 (62.5%) and 3 (37.5%) positive for HAdV respectively. In the control group, all positive samples belonged to the 1–3 years old age group (p-value = 0.02), while in the case group, out of 8 positive samples 5 (62.5%), 2 (25%) and 1 (12.5%) belonged to under 1, 1–3 and 4–5 years old age groups, respectively (Table1).

**Table 1:** Comparison of adenovirus-positive cases frequency based on age and sex between two groups of children with SARI and without any respiratory symptoms

Demographic data Age group (year)			genotyping		Total	Exact McNemar test 2-sided P-value
			positive	negative		
<1		case	5	51	56	
		control	0	54	54	< 0.001
	Total		5	105	110	
1–3		case	2	52	54	
		control	8	42	50	< 0.001
	Total		10	94	104	
4–5		case	1	9	10	
		control	0	16	16	0.004
	Total		1	25	26	

**Fig. 1:**
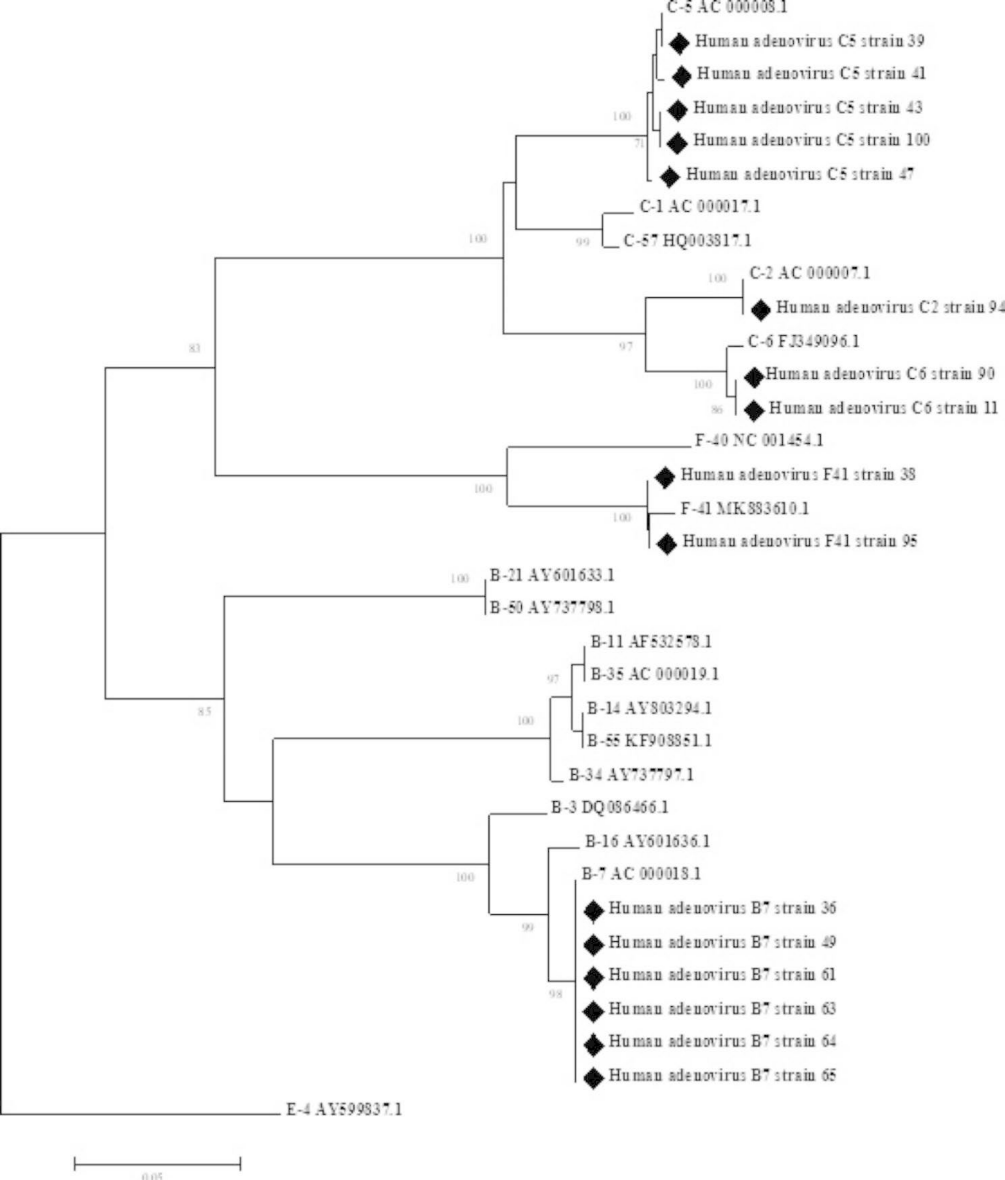
Phylogenetic tree was inferred by using the Neighbor joining method based on the Tamura-Nei model with 1000 bootstrap analyses. A region with 330 nucleotides length from HAdV hexon was sequenced. The accession numbers for reference strains are written next to their types and 16 strains identified in this study (GenBank accession numbers MW558248-MW558263) are marked with ♦

**Fig. 2:**
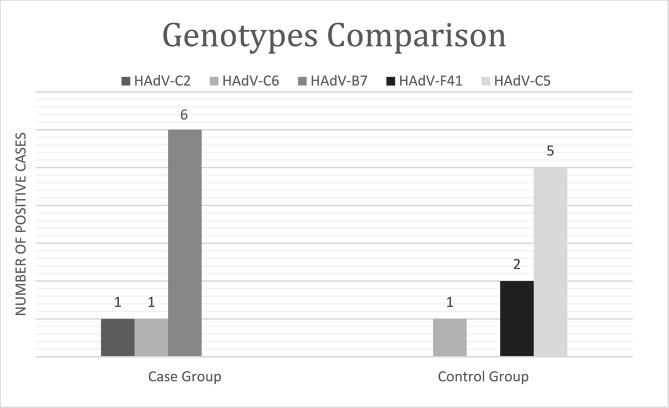
Comparison of adenovirus positive cases based on genotype in two groups: children with SARI**(A)** and without any respiratory symptoms **(B)**

Sequencing was performed for all 16 positive samples. Five different HAdV types were identified indicating that HAdV-B7 was the predominant type, followed by HAdV-C5. By phylogenetic analysis (Fig.1) our strains in the control group matched with HAdV-C5 (62.5%), HAdV-F41 (25%) and HAdV-C6 (12.5%). Whereas, in the case group HAdV-B7 comprised 75% of positive cases (6/8, p-value = 0.02), followed by HAdV-C2 (12.5%,1/8) and HAdV-C6 (12.5%, 1/8) (Fig.2).

The identity between Iranian HAdV strains and reference sequences of the same types is shown in Table2.

**Table 2:** The identity between Iranian adenovirus strains and reference sequences of the same types

Strain name	Type	Identity (%)	GenBank accession number
Strain 36	Human adenovirus B7	100	MW558248
Strain 49	Human adenovirus B7	100	MW558249
Strain 61	Human adenovirus B7	100	MW558250
Strain 63	Human adenovirus B7	100	MW558251
Strain 64	Human adenovirus B7	100	MW558252
Strain 65	Human adenovirus B7	100	MW558253
Strain 39	Human adenovirus C5	100	MW558254
Strain 41	Human adenovirus C5	99.6	MW558256
Strain 43	Human adenovirus C5	99.6	MW558255
Strain 47	Human adenovirus C5	99.6	MW558257
Strain 100	Human adenovirus C5	99.6	MW558261
Strain 90	Human adenovirus C6	98.9	MW558258
Strain 11	Human adenovirus C6	98.9	MW558259
Strain 94	Human adenovirus C2	100	MW558260
Strain 38	Human adenovirus F41	99.3	MW558262
Strain 95	Human adenovirus F41	99.3	MW558263

## Discussion

In the present study, the detection rate and molecular epidemiology of HAdV in oropharyngeal swabs obtained from two groups of children aged five and younger with SARI and without any respiratory symptoms were investigated.

We report that, HAdV-DNA was identified in 8 patients (6.7%) and 8 control samples (6.7%). The predominant type in the case and control groups, were HAdV-B7 and HAdV-C5, respectively. It is known that HAdV-B7 is associated with severe diseases. Within the SARI patients, 56% were < 1 year and also five out of six HAdV-B7 positive cases were < 1 year. This actually indicates that < 1 age group is more important from the SARI point of view.

In a case-control study in Norway, adenoviral infections were detected in 6.1% of children with respiratory tract infections, whereas the control group’s detection rate was 10.5%. The most common types were HAdV-B3, HAdV-C2, and HAdV-C1[13]. Former studies in Iran showed that 14.4% [14], 18.4% [3] and 35.5% [2] of children with SARI were positive for HAdV. In the latter study, the most prevalent species among children with SARI was *HAdV-B* (80.3%), followed by *HAdV-C* (15.5%), *HAdV-D* (2.8%) and *HAdV-E* (1.4%). Besides, HAdV- B14*/*55 and HAdV-B3 were the predominant types [2]. Makvandi et al. detected HAdV-5 (86.3%) and HAdV-2 (13.7%) in children diagnosed with SARI [3]. In Egypt, the prevalence rate of HAdV in hospitalized pediatric patients was 35%, in which *HAdV-B* was the most common (76.2%), followed by *HAdV-C* (19%) and *HAdV-E* (4.8%) [15]. Various studies in China have reported different detection rates, for instance 3.72%, in which the common types were HAdV-C2, HAdV-B3, HAdV-B7 [16], 9.4% where the most prevalent type was HAdV-B7 (34%) [9] and another study with 13.3% HAdV prevalence in children with pneumonia which HAdV-B7 was the main serotype [17].

Some serotypes of HAdVs have been reported to be associated with more severe infections. A study in Taiwan has revealed an association between HAdV-B7 and severe pulmonary complications, respiratory failure, longer hospitalization, long-term pulmonary sequel, as well as intensive-care requirements even among children with no symptoms, in comparison to HAdV-C2 and HAdV-B3 [18]. As shown in some studies, the fatality rate of HAdV-B7 and HAdV-3 infections was higher rather than other types [9, 18–22]. Lai et al. showed that out of 45 severe HAdV infected cases 22 (49%) had HAdV-B7 and also 7 (70%) of fatal cases were due to HAdV-B7 which indicated the mortality rate of HAdV-B7 was 32%[23]. As it was reported by Na Zeng et al. HAdV-B7 infection triggers respiratory failure, severe pneumonia, toxic encephalopathy and lower white blood cell and platelets count. They noticed that in cell culture HAdV-B7 replicates more than HAdV-B3 and also induces aggravated cytokine response provoking severe inflammation in the respiratory tract [24].

In numerous studies, the HAdV detection rate in children with SARI has been reported 2–10% [13]. With a detection rate of 6.7%, our data was in agreement with most of the other studies. A noteworthy point is that HAdV detection in respiratory tracts does not necessarily imply that SARI is due to the presence of this virus. It could be a consequence of HAdV latency and sheddingfor a long time. Many studies have indicated that HAdV, specifically *HAdV-C*, can persist in the lymphocytes of tonsils and adenoids [25]. As mentioned, herein more *HAdV-C* cases were detected in the control group in comparison to the patients. This may be on the grounds that in the control group, where patients do not have respiratory symptoms, adenovirus-DNA presence in oropharyngeal swabs may be a result of a former infection rather than a recent one. Since we detected HAdV-B7 cases in the patients with SARI, contrary to the control group, this data was also consistent with other studies which indicated that HAdV-B7 leads to more severe conditions. It is noticeable that in this study, two cases of HAdV-F41, which is an enteric HAdV, were found in oropharyngeal swabs in the control group. Previously, in a study in Argentina, out of 26 positive HAdV respiratory samples 2 were identified as *HAdV-F*. The underlying reason for detecting *HAdV-F* in the respiratory tract has not been recognized yet. It is assumed that the respiratory tract could either be an entry or replication site for *HAdV-F* [26]. Further research is needed to justify this phenomenon.

As it is evident, HAdV prevalent types alter through the years in different countries. Thus, monitoring the molecular epidemiology of HAdV in different sites and over the years is of high importance.

This study has some limitations we need to highlight. Clinical data on the study subjects such as days of symptoms, clinical presentations, comorbidities, and socioeconomic status were not available. Besides, there was no access to follow-up data for the controls on developed respiratory symptoms after sampling.

## Conclusion

To our knowledge, the present study is the only research which has raised a different viewpoint of HAdV molecular epidemiology where the genotypes were compared in children with and without respiratory symptoms. HAdV prevalence was equally common in cases and controls but different genotypes were detected in these two groups. *HAdV-B*, which has been associated with a more severe condition, was only identified in the case group. Continued surveillance may be helpful regarding control and treatment of adenoviral infections.

## Data Availability

The datasets collected and analyzed during this study are not publicly available due to the National Influenza Center policy but are available from the corresponding author on reasonable request.

## Abbreviations

HAdV: Human adenovirus.
SARI: severe acute respiratory infection.
NIC: National Influenza Center.
PCR: polymerase chain reaction.
